# Supplementary material for: A Serial Cross-Sectional Analysis of the Prevalence, Risk Factors and Geographic Variations of Reduced Visual Acuity in Primary and Secondary Students from 2000 to 2017 in Hong Kong
Source: Int J Environ Res Public Health. 2020 Feb 6;17(3):1023. doi: 10.3390/ijerph17031023 (PMC7036919; doi:10.3390/ijerph17031023)
Supplement: Supplementary file 1 [file ijerph-17-01023-s001.docx]

|  |  |  |  |  |  |  |  |  |  |  |  |  |  |  |  |  |
| --- | --- | --- | --- | --- | --- | --- | --- | --- | --- | --- | --- | --- | --- | --- | --- | --- |
|  | 2000/01 | 2001/02 | 2002/03 | 2003/04 | 2004/05 | 2005/06 | 2006/07 | 2007/08 | 2008/09 | 2010/11 | 2011/12 | 2012/13 | 2013/14 | 2014/15 | 2015/16 | 2016/17 |
| Participation rate (%) | | | | | | | | | | | | | | | | |
| Students | **55.73** | 59.63 | 59.82 | 63.6 | 63.91 | 65.09 | 65.77 | 64.62 | 63.82 | 65.69 | 64.33 | 65.16 | **69.76** | 66.3 | 66.4 | 66.49 |
|  |  |  |  |  |  |  |  |  |  |  |  |  |  |  |  |  |
| Age | | | | | | | | | | | | | | | | |
| Mean | **9.72** | 9.8 | 9.9 | 10.03 | 10.12 | 10.26 | 10.35 | 10.43 | 10.49 | **10.64** | 10.46 | 10.35 | 10.23 | 10.14 | 10.06 | 9.98 |
| Std Dev | **2.49** | 2.51 | 2.53 | 2.55 | 2.53 | 2.54 | 2.57 | 2.59 | 2.63 | **2.73** | 2.71 | 2.72 | 2.75 | 2.75 | 2.71 | 2.66 |
|  |  |  |  |  |  |  |  |  |  |  |  |  |  |  |  |  |
| Sex (%) | | | | | | | | | | | | | | | | |
| Girls | **50.07** | 50.19 | 50.19 | 50.1 | 50.11 | 49.82 | 49.59 | 49.54 | 49.55 | 49.61 | 49.15 | 49.02 | 48.84 | 48.87 | 48.77 | **48.63** |
| Boys | **49.93** | 49.81 | 49.81 | 49.9 | 49.89 | 50.18 | 50.41 | 50.46 | 50.45 | 50.39 | 50.85 | 50.98 | 51.16 | 51.13 | 51.23 | **51.37** |
|  |  |  |  |  |  |  |  |  |  |  |  |  |  |  |  |  |
| Presenting visual acuity (Right) | | | | | | | | | | | | | | | | |
| Mean | **0.229** | 0.23 | 0.227 | 0.228 | 0.229 | 0.225 | 0.222 | 0.222 | 0.222 | 0.225 | 0.226 | 0.16 | 0.156 | 0.166 | 0.171 | **0.173** |
| Std Dev | **0.142** | 0.142 | 0.139 | 0.141 | 0.14 | 0.14 | 0.139 | 0.141 | 0.139 | 0.139 | 0.142 | 0.167 | 0.167 | 0.175 | 0.177 | **0.18** |
|  |  |  |  |  |  |  |  |  |  |  |  |  |  |  |  |  |
| Presenting visual acuity (Left) | | | | | | | | | | | | | | | | |
| Mean | **0.235** | 0.235 | 0.232 | 0.233 | 0.234 | 0.231 | 0.227 | 0.228 | 0.227 | 0.229 | 0.229 | 0.16 | 0.157 | 0.165 | 0.17 | **0.171** |
| Std Dev | **0.136** | 0.136 | 0.133 | 0.135 | 0.134 | 0.134 | 0.133 | 0.135 | 0.133 | 0.133 | 0.136 | 0.164 | 0.164 | 0.171 | 0.173 | **0.175** |
|  |  |  |  |  |  |  |  |  |  |  |  |  |  |  |  |  |
| Usage of visual aids (%) | | | | | | | | | | | | | | | | |
| No | **67.57** | 66.65 | 65.89 | 64.94 | 63.02 | 61.08 | 59.92 | 59.11 | 58 | **57.1** | 58.58 | 59.93 | 61 | 62 | 62.8 | 63.96 |
| Yes | **32.43** | 33.35 | 34.11 | 35.06 | 36.98 | 38.92 | 40.08 | 40.89 | 42 | **42.9** | 41.42 | 40.06 | 39 | 38 | 37.2 | 36.04 |
|  |  |  |  |  |  |  |  |  |  |  |  |  |  |  |  |  |
| School year 2009/10 was excluded because SHS limited the annual appointments in that year for taking part in the Human Swine Influenza Vaccination Programme | | | | | | | | | | | | | | | | |

**Supplementary Table 1.** Demographic information of schoolchildren included in the study.

**Supplementary Table 2.** Missing data pattern of variables of schoolchildren included in the study.

|  |  | Variables included | | | | | |
| --- | --- | --- | --- | --- | --- | --- | --- |
|  |  | Home district | | Presenting visual acuity | | Usage of visual aids | |
|  |  |  |  |  |  |  |  |
| School Year | N (‘000) | n | % | n | % | n | % |
|  |  |  |  |  |  |  |  |
| 2000/01 | 435 | 393 | 0.09 | 504 | 0.12 | 504 | 0.12 |
| 2001/02 | 465 | 372 | 0.08 | 348 | 0.07 | 348 | 0.07 |
| 2002/03 | 464 | 464 | 0.1 | 302 | 0.07 | 302 | 0.07 |
| 2003/04 | 487 | 1160 | 0.24 | 348 | 0.07 | 348 | 0.07 |
| 2004/05 | 478 | 2798 | 0.59 | 317 | 0.07 | 317 | 0.07 |
| 2005/06 | 473 | 883 | 0.19 | 281 | 0.06 | 281 | 0.06 |
| 2006/07 | 469 | 2616 | 0.56 | 253 | 0.05 | 253 | 0.05 |
| 2007/08 | 448 | 2915 | 0.65 | 272 | 0.06 | 272 | 0.06 |
| 2008/09 | 426 | 3253 | 0.76 | 293 | 0.07 | 293 | 0.07 |
| 2009/10 | - | - | - | - | - | - | - |
| 2010/11 | 402 | 2582 | 0.64 | 131 | 0.03 | 131 | 0.03 |
| 2011/12 | 377 | 1495 | 0.4 | 84 | 0.02 | 84 | 0.02 |
| 2012/13 | 367 | 2017 | 0.55 | 88 | 0.02 | 88 | 0.02 |
| 2013/14 | 364 | 1046 | 0.29 | 94 | 0.03 | 94 | 0.03 |
| 2014/15 | 364 | 1253 | 0.34 | 77 | 0.02 | 77 | 0.02 |
| 2015/16 | 364 | 2046 | 0.56 | 86 | 0.02 | 86 | 0.02 |
| 2016/17 | 367 | 5739 | 1.57 | 89 | 0.02 | 89 | 0.02 |
|  |  |  |  |  |  |  |  |
| School year 2009/10 was excluded because SHS limited the annual appointments in that year for taking part in the Human Swine Influenza Vaccination Programme | | | | | | | |

**Supplementary Table 3.** Subgroup analysis of the prevalence of reduced visual acuity, stratified by age and grade.

| **(a) Prevalence, stratified by age** | | | | | | | | | | | | | | | |
| --- | --- | --- | --- | --- | --- | --- | --- | --- | --- | --- | --- | --- | --- | --- | --- |
| School  Year | 6 - 7 | | | 8 - 9 | | | 10 - 11 | | | 12 – 13 | | | 14 - 15 | | |
|  | Estimate | 95% LB | 95% UB | Estimate | 95% LB | 95% UB | Estimate | 95% LB | 95% UB | Estimate | 95% LB | 95% UB | Estimate | 95% LB | 95% UB |
| 2000/01 | **26.08** | **25.76** | **26.40** | 38.89 | 38.53 | 39.25 | 52.44 | 52.01 | 52.88 | 61.48 | 60.91 | 62.05 | **66.22** | **65.37** | **67.07** |
| 2001/02 | 26.39 | 26.08 | 26.71 | 38.81 | 38.46 | 39.16 | 52.38 | 51.96 | 52.80 | 61.78 | 61.24 | 62.32 | 66.87 | 66.07 | 67.67 |
| 2002/03 | 24.74 | 24.43 | 25.05 | 38.17 | 37.83 | 38.52 | 51.88 | 51.46 | 52.29 | 61.66 | 61.12 | 62.20 | 67.33 | 66.58 | 68.09 |
| 2003/04 | 25.67 | 25.34 | 25.99 | 39.24 | 38.90 | 39.59 | 52.73 | 52.32 | 53.14 | 62.29 | 61.76 | 62.81 | 67.79 | 67.10 | 68.48 |
| 2004/05 | 25.95 | 25.61 | 26.29 | 41.19 | 40.83 | 41.55 | 54.49 | 54.08 | 54.90 | 63.29 | 62.78 | 63.81 | 68.85 | 68.15 | 69.55 |
| 2005/06 | 26.76 | 26.39 | 27.12 | 42.21 | 41.83 | 42.58 | 55.62 | 55.20 | 56.03 | 64.07 | 63.56 | 64.58 | 68.85 | 68.18 | 69.52 |
| 2006/07 | 26.79 | 26.43 | 27.15 | 42.85 | 42.46 | 43.25 | 56.01 | 55.59 | 56.43 | 64.47 | 63.97 | 64.97 | 68.70 | 68.05 | 69.36 |
| 2007/08 | 27.72 | 27.34 | 28.10 | 43.53 | 43.11 | 43.94 | 57.04 | 56.60 | 57.48 | 64.85 | 64.35 | 65.35 | 69.06 | 68.41 | 69.70 |
| 2008/09 | 29.00 | 28.59 | 29.40 | 44.47 | 44.04 | 44.90 | 57.96 | 57.49 | 58.43 | 65.88 | 65.37 | 66.40 | 69.74 | 69.10 | 70.38 |
| 2009/10 |  |  |  |  |  |  |  |  |  |  |  |  |  |  |  |
| 2010/11 | 29.97 | 29.55 | 30.39 | 46.00 | 45.53 | 46.46 | 58.06 | 57.56 | 58.55 | 65.72 | 65.17 | 66.27 | 69.55 | 68.97 | 70.13 |
| 2011/12 | **31.13** | **30.70** | **31.55** | 46.64 | 46.17 | 47.12 | 58.29 | 57.78 | 58.80 | 65.12 | 64.55 | 65.69 | **69.46** | **68.81** | **70.10** |
| 2012/13 | 23.96 | 23.60 | 24.32 | 43.84 | 43.37 | 44.30 | 56.78 | 56.26 | 57.30 | 63.86 | 63.30 | 64.42 | 68.22 | 67.55 | 68.89 |
| 2013/14 | 23.55 | 23.21 | 23.89 | 42.75 | 42.30 | 43.20 | 56.46 | 55.93 | 56.99 | 63.97 | 63.39 | 64.54 | 67.85 | 67.16 | 68.54 |
| 2014/15 | 24.20 | 23.86 | 24.54 | 42.82 | 42.38 | 43.26 | 56.92 | 56.38 | 57.46 | 64.48 | 63.87 | 65.08 | 68.17 | 67.48 | 68.86 |
| 2015/16 | 24.57 | 24.22 | 24.91 | 42.04 | 41.61 | 42.46 | 57.32 | 56.79 | 57.85 | 64.67 | 64.05 | 65.29 | 68.61 | 67.90 | 69.32 |
| 2016/17 | **24.47** | **24.13** | **24.81** | 41.09 | 40.68 | 41.50 | 56.18 | 55.67 | 56.70 | 65.18 | 64.55 | 65.81 | **68.89** | **68.14** | **69.63** |
|  |  |  |  |  |  |  |  |  |  |  |  |  |  |  |  |
| Mann-Kendall Trend Test | | | | | | | | | | | | | | | |
| Time series | | tau | p-value |  | tau | p-value |  | tau | p-value |  | tau | p-value |  | tau | p-value |
|  | |  |  |  |  |  |  |  |  |  |  |  |  |  |  |
| Overall |  |  |  |  |  |  |  |  |  |  |  |  |  |  |  |
| 2000/01 – 2016/17 | | -0.05 | 0.822 |  | 0.367 | 0.053 |  | **0.55** | **0.003** |  | **0.567** | **0.003** |  | 0.343 | 0.071 |
|  |  |  |  |  |  |  |  |  |  |  |  |  |  |  |  |
| Breakdown | |  |  |  |  |  |  |  |  |  |  |  |  |  |  |
| 2000/01 – 2011/12 | | **0.782** | **0.001** |  | **0.891** | **<0.001** |  | **0.891** | **<0.001** |  | **0.855** | **<0.001** |  | **0.807** | **<0.001** |
| 2012/13 – 2016/17 | | 0.6 | 0.221 |  | -0.8 | 0.086 |  | 0 | 1 |  | **1** | **0.027** |  | 0.6 | 0.221 |
|  |  |  |  |  |  |  |  |  |  |  |  |  |  |  |  |
| (b) Prevalence, stratified by grade | | | | | | | | | | | | | | | |
|  |  |  |  |  |  |  |  |  |  |  |  |  |  |  |  |
| School  Year | P1 - P2 | | | P3 - P4 | | | P5 - P6 | | | S1 - S2 | | | S3 - S4 | | |
|  | Estimate | 95% LB | 95% UB | Estimate | 95% LB | 95% UB | Estimate | 95% LB | 95% UB | Estimate | 95% LB | 95% UB | Estimate | 95% LB | 95% UB |
| 2000/01 | **27.40** | **27.11** | **27.70** | 41.56 | 41.19 | 41.92 | 55.38 | 54.93 | 55.83 | 65.26 | 64.62 | 65.90 | **69.05** | **68.06** | **70.05** |
| 2001/02 | 27.97 | 27.68 | 28.27 | 41.23 | 40.88 | 41.59 | 55.31 | 54.88 | 55.74 | 65.04 | 64.45 | 65.63 | 69.77 | 68.84 | 70.70 |
| 2002/03 | 26.71 | 26.42 | 27.01 | 40.64 | 40.29 | 40.99 | 54.62 | 54.19 | 55.05 | 64.57 | 63.99 | 65.15 | 69.87 | 69.01 | 70.74 |
| 2003/04 | 27.83 | 27.52 | 28.13 | 41.73 | 41.38 | 42.08 | 55.28 | 54.86 | 55.70 | 64.93 | 64.38 | 65.48 | 69.83 | 69.05 | 70.61 |
| 2004/05 | 28.64 | 28.32 | 28.96 | 43.81 | 43.44 | 44.17 | 56.71 | 56.29 | 57.13 | 65.44 | 64.90 | 65.99 | 70.43 | 69.67 | 71.20 |
| 2005/06 | 29.22 | 28.88 | 29.56 | 45.23 | 44.85 | 45.61 | 57.82 | 57.39 | 58.24 | 65.70 | 65.17 | 66.23 | 70.28 | 69.56 | 71.00 |
| 2006/07 | 29.52 | 29.17 | 29.87 | 45.68 | 45.28 | 46.07 | 58.34 | 57.91 | 58.77 | 65.89 | 65.37 | 66.41 | 70.15 | 69.45 | 70.84 |
| 2007/08 | 30.33 | 29.97 | 30.69 | 46.62 | 46.20 | 47.04 | 59.35 | 58.91 | 59.80 | 66.29 | 65.77 | 66.80 | 70.29 | 69.60 | 70.98 |
| 2008/09 | 31.62 | 31.24 | 32.01 | 47.25 | 46.82 | 47.68 | 60.02 | 59.55 | 60.49 | 67.07 | 66.54 | 67.60 | 70.93 | 70.25 | 71.61 |
| 2009/10 |  |  |  |  |  |  |  |  |  |  |  |  |  |  |  |
| 2010/11 | 32.69 | 32.28 | 33.10 | 48.56 | 48.10 | 49.02 | 60.01 | 59.51 | 60.50 | 66.87 | 66.32 | 67.42 | 69.77 | 69.15 | 70.40 |
| 2011/12 | **33.76** | **33.35** | **34.18** | 49.19 | 48.71 | 49.67 | 59.93 | 59.43 | 60.44 | 66.10 | 65.53 | 66.67 | **70.48** | **69.81** | **71.16** |
| 2012/13 | 27.37 | 27.00 | 27.73 | 46.70 | 46.23 | 47.17 | 58.48 | 57.96 | 59.00 | 64.76 | 64.19 | 65.33 | 69.47 | 68.78 | 70.16 |
| 2013/14 | 26.47 | 26.12 | 26.81 | 45.89 | 45.42 | 46.36 | 58.31 | 57.77 | 58.84 | 64.73 | 64.15 | 65.31 | 68.95 | 68.24 | 69.66 |
| 2014/15 | 27.46 | 27.11 | 27.80 | 45.77 | 45.32 | 46.23 | 59.06 | 58.51 | 59.61 | 65.27 | 64.66 | 65.87 | 68.98 | 68.26 | 69.70 |
| 2015/16 | 27.43 | 27.09 | 27.77 | 45.20 | 44.76 | 45.65 | 59.36 | 58.81 | 59.91 | 65.57 | 64.95 | 66.20 | 69.44 | 68.70 | 70.17 |
| 2016/17 | **27.36** | **27.01** | **27.70** | 44.08 | 43.65 | 44.50 | 58.48 | 57.95 | 59.02 | 66.12 | 65.48 | 66.76 | **69.44** | **68.67** | **70.20** |
|  |  |  |  |  |  |  |  |  |  |  |  |  |  |  |  |
| Mann-Kendall Trend Test | | | | | | | | | | | | | | | |
| Time series | | tau | p-value |  | tau | p-value |  | tau | p-value |  | tau | p-value |  | tau | p-value |
|  |  |  |  |  |  |  |  |  |  |  |  |  |  |  |  |
| Overall | |  |  |  |  |  |  |  |  |  |  |  |  |  |  |
| 2000/01 – 2016/17 | | 0.05 | 0.822 |  | **0.4** | **0.034** |  | **0.477** | **0.012** |  | 0.233 | 0.224 |  | -0.168 | 0.391 |
|  |  |  |  |  |  |  |  |  |  |  |  |  |  |  |  |
| Breakdown | |  |  |  |  |  |  |  |  |  |  |  |  |  |  |
| 2000/01 – 2011/12 | | **0.891** | **<0.001** |  | **0.891** | **<0.001** |  | **0.709** | **0.003** |  | **0.673** | **0.005** |  | **0.514** | **0.035** |
| 2012/13 – 2016/17 | | 0 | 1 |  | **-1** | **0.027** |  | 0.316 | 0.613 |  | 0.8 | 0.086 |  | 0.105 | 1 |
|  |  |  |  |  |  |  |  |  |  |  |  |  |  |  |  |
| LB, lower bound; UB, upper bound; SHS, Student Health Service | | | | | | | | | | | | | | | |
| School year 2009/10 was excluded because SHS limited the annual appointments in that year for taking part in the Human Swine Influenza Vaccination Programme | | | | | | | | | | | | | | | |

**Supplementary Table 4.** Subgroup analysis of the odds ratios (girls relative to boys) of reduced visual acuity, stratified by age and grade.

| **(a) Odds ratio (girls relative to boys), stratified by age** | | | | | | | | | | | | | | | |
| --- | --- | --- | --- | --- | --- | --- | --- | --- | --- | --- | --- | --- | --- | --- | --- |
|  |  |  |  |  |  |  |  |  |  |  |  |  |  |  |  |
| School  Year | 6 - 7 | | | 8 - 9 | | | 10 - 11 | | | 12 - 13 | | | 14 - 15 | | |
|  | Estimate | 95% LB | 95% UB | Estimate | 95% LB | 95% UB | Estimate | 95% LB | 95% UB | Estimate | 95% LB | 95% UB | Estimate | 95% LB | 95% UB |
| 2000/01 | **0.937** | **0.911** | **0.964** | **1.033** | **1.008** | **1.057** | 1.125 | 1.098 | 1.152 | 1.207 | 1.171 | 1.243 | **1.175** | **1.124** | **1.228** |
| 2001/02 | 0.942 | 0.916 | 0.968 | 1.054 | 1.030 | 1.079 | 1.120 | 1.095 | 1.147 | 1.208 | 1.174 | 1.243 | 1.207 | 1.158 | 1.257 |
| 2002/03 | 0.950 | 0.923 | 0.979 | 1.013 | 0.990 | 1.037 | 1.143 | 1.116 | 1.169 | 1.166 | 1.134 | 1.200 | 1.165 | 1.120 | 1.211 |
| 2003/04 | 0.942 | 0.914 | 0.970 | 1.035 | 1.012 | 1.059 | 1.163 | 1.137 | 1.190 | 1.165 | 1.134 | 1.197 | 1.158 | 1.118 | 1.201 |
| 2004/05 | 0.960 | 0.931 | 0.990 | 1.017 | 0.994 | 1.041 | 1.168 | 1.142 | 1.195 | 1.230 | 1.197 | 1.264 | 1.155 | 1.114 | 1.198 |
| 2005/06 | 0.962 | 0.931 | 0.993 | 1.056 | 1.031 | 1.081 | 1.155 | 1.130 | 1.181 | 1.257 | 1.224 | 1.290 | 1.147 | 1.108 | 1.188 |
| 2006/07 | 0.934 | 0.905 | 0.964 | 1.061 | 1.035 | 1.087 | 1.167 | 1.141 | 1.194 | 1.229 | 1.197 | 1.261 | 1.203 | 1.163 | 1.244 |
| 2007/08 | 0.965 | 0.934 | 0.997 | 1.021 | 0.996 | 1.048 | 1.180 | 1.152 | 1.208 | 1.203 | 1.172 | 1.235 | 1.200 | 1.160 | 1.241 |
| 2008/09 | 0.935 | 0.904 | 0.966 | 1.034 | 1.008 | 1.061 | 1.163 | 1.134 | 1.193 | 1.195 | 1.163 | 1.228 | 1.181 | 1.143 | 1.221 |
| 2009/10 |  |  |  |  |  |  |  |  |  |  |  |  |  |  |  |
| 2010/11 | **0.928** | **0.898** | **0.960** | 1.012 | 0.984 | 1.040 | **1.126** | **1.097** | **1.156** | 1.201 | 1.167 | 1.236 | **1.157** | **1.123** | **1.193** |
| 2011/12 | 0.914 | 0.884 | 0.944 | 1.002 | 0.974 | 1.030 | 1.115 | 1.086 | 1.146 | 1.146 | 1.113 | 1.181 | 1.218 | 1.178 | 1.260 |
| 2012/13 | 0.895 | 0.865 | 0.926 | 0.961 | 0.934 | 0.989 | 1.121 | 1.090 | 1.152 | 1.162 | 1.128 | 1.196 | 1.207 | 1.165 | 1.250 |
| 2013/14 | 0.909 | 0.880 | 0.940 | 0.989 | 0.962 | 1.016 | 1.071 | 1.041 | 1.102 | 1.161 | 1.127 | 1.197 | 1.154 | 1.113 | 1.196 |
| 2014/15 | 0.919 | 0.889 | 0.949 | 0.997 | 0.970 | 1.024 | 1.082 | 1.052 | 1.114 | 1.196 | 1.159 | 1.235 | 1.225 | 1.182 | 1.270 |
| 2015/16 | 0.943 | 0.913 | 0.974 | 1.009 | 0.982 | 1.036 | 1.115 | 1.084 | 1.147 | 1.192 | 1.154 | 1.231 | 1.242 | 1.196 | 1.289 |
| 2016/17 | 0.937 | 0.907 | 0.968 | 1.018 | 0.992 | 1.045 | 1.148 | 1.116 | 1.180 | 1.234 | 1.194 | 1.275 | 1.272 | 1.224 | 1.323 |
|  |  |  |  |  |  |  |  |  |  |  |  |  |  |  |  |
| (b) Odds ratio (girls relative to boys), stratified by grade | | | | | | | | | | | | | | | |
|  |  |  |  |  |  |  |  |  |  |  |  |  |  |  |  |
| School  Year | P1 - P2 | | | P3 - P4 | | | P5 - P6 | | | S1 - S2 | | | S3 - S4 | | |
|  | Estimate | 95% LB | 95% UB | Estimate | 95% LB | 95% UB | Estimate | 95% LB | 95% UB | Estimate | 95% LB | 95% UB | Estimate | 95% LB | 95% UB |
| 2000/01 | **0.957** | **0.933** | **0.982** | **1.046** | **1.023** | **1.070** | 1.159 | 1.132 | 1.188 | 1.182 | 1.143 | 1.222 | **1.169** | **1.111** | **1.231** |
| 2001/02 | 0.952 | 0.928 | 0.975 | 1.065 | 1.042 | 1.089 | 1.141 | 1.115 | 1.168 | 1.209 | 1.172 | 1.246 | 1.136 | 1.082 | 1.192 |
| 2002/03 | 0.957 | 0.933 | 0.982 | 1.029 | 1.006 | 1.053 | 1.150 | 1.124 | 1.177 | 1.169 | 1.135 | 1.205 | 1.127 | 1.078 | 1.180 |
| 2003/04 | 0.958 | 0.933 | 0.983 | 1.050 | 1.027 | 1.073 | 1.167 | 1.141 | 1.194 | 1.180 | 1.147 | 1.214 | 1.152 | 1.106 | 1.199 |
| 2004/05 | 0.966 | 0.940 | 0.992 | 1.038 | 1.016 | 1.062 | 1.187 | 1.160 | 1.214 | 1.210 | 1.176 | 1.245 | 1.145 | 1.100 | 1.191 |
| 2005/06 | 0.963 | 0.937 | 0.990 | 1.075 | 1.051 | 1.100 | 1.170 | 1.144 | 1.197 | 1.250 | 1.215 | 1.284 | 1.148 | 1.105 | 1.192 |
| 2006/07 | 0.952 | 0.926 | 0.979 | 1.085 | 1.060 | 1.111 | 1.177 | 1.150 | 1.204 | 1.225 | 1.192 | 1.258 | 1.181 | 1.139 | 1.224 |
| 2007/08 | 0.964 | 0.937 | 0.992 | 1.049 | 1.023 | 1.075 | 1.194 | 1.166 | 1.223 | 1.183 | 1.152 | 1.215 | 1.178 | 1.136 | 1.221 |
| 2008/09 | 0.952 | 0.924 | 0.980 | 1.040 | 1.014 | 1.066 | 1.185 | 1.156 | 1.215 | 1.178 | 1.146 | 1.211 | 1.197 | 1.155 | 1.240 |
| 2009/10 |  |  |  |  |  |  |  |  |  |  |  |  |  |  |  |
| 2010/11 | 0.938 | 0.910 | 0.966 | 1.033 | 1.006 | 1.061 | 1.132 | 1.103 | 1.162 | 1.208 | 1.174 | 1.244 | 1.143 | 1.106 | 1.181 |
| 2011/12 | **0.929** | **0.902** | **0.957** | 1.008 | 0.981 | 1.036 | **1.124** | **1.094** | **1.155** | 1.164 | 1.130 | 1.199 | **1.215** | **1.173** | **1.259** |
| 2012/13 | 0.907 | 0.880 | 0.936 | 0.990 | 0.963 | 1.018 | 1.125 | 1.095 | 1.157 | 1.160 | 1.127 | 1.195 | 1.212 | 1.169 | 1.256 |
| 2013/14 | 0.914 | 0.886 | 0.942 | 1.002 | 0.975 | 1.030 | 1.077 | 1.046 | 1.108 | 1.170 | 1.135 | 1.206 | 1.162 | 1.120 | 1.206 |
| 2014/15 | 0.929 | 0.902 | 0.957 | 1.001 | 0.974 | 1.029 | 1.090 | 1.059 | 1.122 | 1.215 | 1.177 | 1.254 | 1.216 | 1.171 | 1.262 |
| 2015/16 | 0.939 | 0.912 | 0.967 | 1.024 | 0.997 | 1.051 | 1.129 | 1.097 | 1.163 | 1.199 | 1.161 | 1.239 | 1.214 | 1.169 | 1.262 |
| 2016/17 | 0.964 | 0.936 | 0.993 | 1.020 | 0.994 | 1.046 | 1.157 | 1.124 | 1.190 | 1.239 | 1.198 | 1.280 | 1.299 | 1.249 | 1.352 |
|  |  |  |  |  |  |  |  |  |  |  |  |  |  |  |  |
| LB, lower bound; UB, upper bound; SHS, Student Health Service | | | | | | | | | | | | | | | |
| School year 2009/10 was excluded because SHS limited the annual appointments in that year for taking part in the Human Swine Influenza Vaccination Programme | | | | | | | | | | | | | | | |

**Supplementary Table 5.** Factors associated with reduced visual acuity in the schoolchildren of Hong Kong during 2000/01-03/04, 2004/05-07/08, 2008/09-12/13 and 2013/14-16/17: Univariate logistic regression before adjusting for age and sex.

|  |  | 2000/01 - 03/04 | | | 2004/05 - 07/08 | | | 2008/09 - 12/13 | | | 2013/14 - 16/17 | | |
| --- | --- | --- | --- | --- | --- | --- | --- | --- | --- | --- | --- | --- | --- |
|  |  |  |  |  |  |  |  |  |  |  |  |  |  |
|  |  | OR | 95% LB | 95% UB | OR | 95% LB | 95% UB | OR | 95% LB | 95% UB | OR | 95% LB | 95% UB |
|  |  |  |  |  |  |  |  |  |  |  |  |  |  |
| age |  | **1.251** | **1.249** | **1.252** | 1.252 | 1.250 | 1.253 | 1.240 | 1.238 | 1.241 | **1.267** | **1.265** | **1.268** |
|  |  |  |  |  |  |  |  |  |  |  |  |  |  |
| Sex | Boys (ref) |  |  |  |  |  |  |  |  |  |  |  |  |
|  | Girls | 1.098 | 1.091 | 1.105 | 1.112 | 1.106 | 1.119 | 1.082 | 1.076 | 1.089 | 1.089 | 1.082 | 1.097 |
|  |  |  |  |  |  |  |  |  |  |  |  |  |  |
| School Type | Primary School (ref) |  |  |  |  |  |  |  |  |  |  |  |  |
|  | Secondary School | **2.898** | **2.875** | **2.920** | 2.712 | 2.694 | 2.731 | 2.522 | 2.504 | 2.540 | **2.696** | **2.676** | **2.716** |
|  |  |  |  |  |  |  |  |  |  |  |  |  |  |
| School District | Central and Western (ref) |  |  |  |  |  |  |  |  |  |  |  |  |
|  | Wan Chai | 0.944 | 0.923 | 0.966 | 0.922 | 0.902 | 0.943 | 0.924 | 0.902 | 0.946 | 0.925 | 0.903 | 0.948 |
|  | Eastern | 0.851 | 0.835 | 0.868 | 0.986 | 0.967 | 1.005 | 1.105 | 1.081 | 1.128 | 1.059 | 1.036 | 1.082 |
|  | Southern | 0.866 | 0.844 | 0.888 | 0.841 | 0.822 | 0.862 | 0.888 | 0.867 | 0.911 | 0.916 | 0.893 | 0.940 |
|  | Yau Tsim Mong | 0.789 | 0.772 | 0.806 | 0.820 | 0.803 | 0.838 | 0.848 | 0.829 | 0.868 | 0.908 | 0.887 | 0.930 |
|  | Sham Shui Po | 0.700 | 0.686 | 0.715 | 0.735 | 0.720 | 0.750 | 0.829 | 0.812 | 0.848 | 0.902 | 0.882 | 0.922 |
|  | Kowloon City | 0.903 | 0.887 | 0.920 | 0.891 | 0.874 | 0.908 | 0.871 | 0.853 | 0.889 | 0.905 | 0.887 | 0.924 |
|  | Wong Tai Sin | 0.621 | 0.609 | 0.633 | 0.673 | 0.660 | 0.686 | 0.832 | 0.814 | 0.850 | 0.928 | 0.907 | 0.949 |
|  | Kwun Tong | 0.665 | 0.653 | 0.678 | 0.709 | 0.695 | 0.723 | 0.827 | 0.810 | 0.845 | 0.894 | 0.875 | 0.913 |
|  | Tsuen Wan | 0.627 | 0.613 | 0.642 | 0.642 | 0.628 | 0.657 | 0.778 | 0.759 | 0.797 | 0.868 | 0.847 | 0.890 |
|  | Tuen Mun | 0.594 | 0.583 | 0.606 | 0.680 | 0.668 | 0.694 | 0.745 | 0.729 | 0.762 | 0.748 | 0.732 | 0.765 |
|  | Yuen Long | 0.559 | 0.548 | 0.569 | 0.674 | 0.662 | 0.687 | 0.838 | 0.821 | 0.855 | 0.761 | 0.745 | 0.777 |
|  | North | 0.637 | 0.624 | 0.650 | 0.666 | 0.653 | 0.680 | 0.690 | 0.675 | 0.706 | 0.698 | 0.682 | 0.714 |
|  | Tai Po | 0.610 | 0.597 | 0.623 | 0.705 | 0.690 | 0.720 | 0.755 | 0.737 | 0.773 | 0.707 | 0.690 | 0.724 |
|  | Sai Kung | 0.696 | 0.681 | 0.712 | 0.726 | 0.711 | 0.741 | 0.938 | 0.917 | 0.959 | 0.994 | 0.971 | 1.017 |
|  | Sha Tin | 0.745 | 0.731 | 0.759 | 0.799 | 0.784 | 0.814 | 0.837 | 0.820 | 0.854 | 0.857 | 0.839 | 0.875 |
|  | Kwai Tsing | 0.747 | 0.732 | 0.762 | 0.754 | 0.739 | 0.770 | 0.857 | 0.838 | 0.876 | 0.929 | 0.908 | 0.950 |
|  | Islands | 0.559 | 0.539 | 0.579 | 0.603 | 0.586 | 0.621 | 0.689 | 0.669 | 0.711 | 0.679 | 0.657 | 0.701 |
|  |  |  |  |  |  |  |  |  |  |  |  |  |  |
| Grade of Student | P1 (ref) |  |  |  |  |  |  |  |  |  |  |  |  |
|  | P2 | 1.402 | 1.384 | 1.420 | 1.530 | 1.509 | 1.552 | 1.578 | 1.554 | 1.602 | 1.682 | 1.657 | 1.707 |
|  | P3 | 1.944 | 1.920 | 1.969 | 2.208 | 2.178 | 2.238 | 2.284 | 2.251 | 2.317 | 2.547 | 2.510 | 2.584 |
|  | P4 | 2.650 | 2.617 | 2.683 | 3.025 | 2.985 | 3.065 | 3.111 | 3.066 | 3.157 | 3.674 | 3.621 | 3.728 |
|  | P5 | 3.499 | 3.455 | 3.543 | 3.872 | 3.821 | 3.924 | 3.834 | 3.779 | 3.889 | 4.718 | 4.649 | 4.789 |
|  | P6 | 4.474 | 4.416 | 4.532 | 4.835 | 4.770 | 4.900 | 4.808 | 4.738 | 4.878 | 5.986 | 5.896 | 6.077 |
|  | S1 | 5.619 | 5.540 | 5.699 | 5.710 | 5.629 | 5.791 | 5.387 | 5.307 | 5.467 | 6.670 | 6.568 | 6.775 |
|  | S2 | 6.477 | 6.376 | 6.579 | 6.443 | 6.348 | 6.541 | 6.082 | 5.989 | 6.177 | 7.467 | 7.348 | 7.588 |
|  | S3 | 7.278 | 7.142 | 7.417 | 7.198 | 7.077 | 7.321 | 6.555 | 6.447 | 6.664 | 7.891 | 7.756 | 8.028 |
|  | S4 | **7.930** | **7.732** | **8.134** | 7.815 | 7.649 | 7.985 | 7.356 | 7.209 | 7.506 | **8.864** | **8.673** | **9.059** |
|  |  |  |  |  |  |  |  |  |  |  |  |  |  |
| Student Health Service Centre | Chai Wan (ref) |  |  |  |  |  |  |  |  |  |  |  |  |
|  | Kowloon Bay | 0.826 | 0.814 | 0.839 | 0.788 | 0.776 | 0.799 | 0.798 | 0.785 | 0.810 | 0.863 | 0.849 | 0.877 |
|  | Kowloon City LC | 0.934 | 0.921 | 0.947 | 0.854 | 0.843 | 0.866 | 0.765 | 0.753 | 0.776 | 0.826 | 0.813 | 0.840 |
|  | Lam Tin | 0.792 | 0.781 | 0.804 | 0.743 | 0.732 | 0.753 | 0.835 | 0.823 | 0.847 | 0.925 | 0.911 | 0.939 |
|  | South Kwai Chung | 0.786 | 0.775 | 0.798 | 0.720 | 0.710 | 0.730 | 0.755 | 0.744 | 0.766 | 0.850 | 0.837 | 0.864 |
|  | Sha Tin | 0.826 | 0.813 | 0.838 | 0.797 | 0.785 | 0.808 | 0.753 | 0.741 | 0.764 | 0.802 | 0.789 | 0.815 |
|  | Tai Po | 0.688 | 0.676 | 0.701 | 0.709 | 0.697 | 0.722 | 0.687 | 0.674 | 0.701 | 0.668 | 0.654 | 0.681 |
|  | Shek Wu Hui | 0.737 | 0.725 | 0.749 | 0.679 | 0.668 | 0.690 | 0.634 | 0.623 | 0.645 | 0.666 | 0.654 | 0.678 |
|  | Tuen Mun | 0.667 | 0.657 | 0.677 | 0.684 | 0.674 | 0.694 | 0.680 | 0.669 | 0.691 | 0.710 | 0.697 | 0.722 |
|  | Western | 1.002 | 0.988 | 1.017 | 0.879 | 0.867 | 0.892 | 0.807 | 0.795 | 0.819 | 0.847 | 0.834 | 0.861 |
|  | TWS Wu York Yu | 0.734 | 0.723 | 0.745 | 0.698 | 0.688 | 0.708 | 0.747 | 0.736 | 0.759 | 0.891 | 0.876 | 0.905 |
|  | Yuen Long | 0.627 | 0.618 | 0.637 | 0.683 | 0.673 | 0.693 | 0.767 | 0.756 | 0.779 | 0.721 | 0.710 | 0.733 |
|  |  |  |  |  |  |  |  |  |  |  |  |  |  |
| Type of Housing | Public rental housing (ref) |  |  |  |  |  |  |  |  |  |  |  |  |
|  | Subsidized home ownership flats | **1.133** | **1.122** | **1.145** | 1.176 | 1.164 | 1.187 | 1.157 | 1.145 | 1.170 | **1.101** | **1.087** | **1.115** |
|  | Private Housing | **1.270** | **1.260** | **1.280** | 1.228 | 1.220 | 1.237 | 1.065 | 1.057 | 1.073 | **0.952** | **0.944** | **0.960** |
|  | Villas/Bungalows/Modern Village Houses | **0.717** | **0.703** | **0.732** | 0.734 | 0.721 | 0.747 | 0.722 | 0.709 | 0.735 | **0.698** | **0.685** | **0.710** |
|  | Squatter/Temp. Housing Area/Stone Hut | **0.645** | **0.626** | **0.664** | 0.753 | 0.728 | 0.779 | 0.756 | 0.723 | 0.790 | **0.772** | **0.729** | **0.817** |
|  |  |  |  |  |  |  |  |  |  |  |  |  |  |
| Home District | Central & Western (ref) |  |  |  |  |  |  |  |  |  |  |  |  |
|  | Wan Chai | 0.943 | 0.908 | 0.979 | 0.908 | 0.880 | 0.937 | 0.853 | 0.825 | 0.883 | 0.860 | 0.831 | 0.891 |
|  | Eastern | 0.941 | 0.925 | 0.957 | 1.071 | 1.051 | 1.091 | 1.108 | 1.085 | 1.132 | 1.071 | 1.046 | 1.096 |
|  | Southern | 0.860 | 0.839 | 0.882 | 0.909 | 0.889 | 0.931 | 0.928 | 0.905 | 0.952 | 0.968 | 0.942 | 0.995 |
|  | Yau Tsim Mong | 0.854 | 0.836 | 0.872 | 0.879 | 0.859 | 0.899 | 0.848 | 0.827 | 0.870 | 0.898 | 0.874 | 0.922 |
|  | Sham Shui Po | 0.780 | 0.765 | 0.796 | 0.804 | 0.788 | 0.822 | 0.904 | 0.884 | 0.926 | 0.946 | 0.923 | 0.970 |
|  | Kowloon City | 0.902 | 0.885 | 0.919 | 0.941 | 0.922 | 0.961 | 0.911 | 0.891 | 0.932 | 0.938 | 0.916 | 0.961 |
|  | Wong Tai Sin | 0.736 | 0.723 | 0.749 | 0.788 | 0.773 | 0.804 | 0.899 | 0.879 | 0.919 | 1.028 | 1.003 | 1.054 |
|  | Kwun Tong | 0.736 | 0.724 | 0.748 | 0.790 | 0.775 | 0.805 | 0.867 | 0.849 | 0.885 | 0.944 | 0.923 | 0.966 |
|  | Tsuen Wan | 0.756 | 0.741 | 0.771 | 0.768 | 0.751 | 0.785 | 0.865 | 0.844 | 0.887 | 0.902 | 0.879 | 0.926 |
|  | Tuen Mun | 0.634 | 0.623 | 0.645 | 0.725 | 0.711 | 0.739 | 0.768 | 0.751 | 0.785 | 0.805 | 0.786 | 0.825 |
|  | Yuen Long | 0.603 | 0.593 | 0.613 | 0.728 | 0.715 | 0.742 | 0.855 | 0.837 | 0.873 | 0.826 | 0.808 | 0.845 |
|  | North | 0.695 | 0.682 | 0.707 | 0.730 | 0.715 | 0.745 | 0.719 | 0.703 | 0.736 | 0.844 | 0.823 | 0.866 |
|  | Tai Po | 0.662 | 0.649 | 0.674 | 0.771 | 0.755 | 0.788 | 0.785 | 0.766 | 0.804 | 0.761 | 0.741 | 0.782 |
|  | Sai Kung | 0.761 | 0.747 | 0.775 | 0.803 | 0.787 | 0.819 | 0.934 | 0.913 | 0.955 | 0.988 | 0.965 | 1.012 |
|  | Sha Tin | 0.804 | 0.791 | 0.817 | 0.852 | 0.836 | 0.868 | 0.852 | 0.834 | 0.870 | 0.885 | 0.865 | 0.906 |
|  | Kwai Tsing | 0.769 | 0.755 | 0.783 | 0.790 | 0.774 | 0.805 | 0.834 | 0.816 | 0.852 | 0.943 | 0.921 | 0.965 |
|  | Islands | 0.682 | 0.658 | 0.708 | 0.677 | 0.659 | 0.696 | 0.695 | 0.675 | 0.716 | 0.721 | 0.699 | 0.744 |
|  |  |  |  |  |  |  |  |  |  |  |  |  |  |
| LB, lower bound; UB, upper bound; SHS, Student Health Service | | | | | | | | | | | | | |
| School year 2009/10 was excluded because SHS limited the annual appointments in that year for taking part in the Human Swine Influenza Vaccination Programme | | | | | | | | | | | | | |

**Supplementary Table 6.** Factors associated with reduced visual acuity in the schoolchildren of Hong Kong during 2000/01-03/04, 2004/05-07/08, 2008/09-12/13 and 2013/14-16/17: Univariate logistic regression after adjusting for age and sex.

|  |  | 2000/01 - 03/04 | | | 2004/05 - 07/08 | | | 2008/09 - 12/13 | | | 2013/14 - 16/17 | | |
| --- | --- | --- | --- | --- | --- | --- | --- | --- | --- | --- | --- | --- | --- |
|  |  |  |  |  |  |  |  |  |  |  |  |  |  |
|  |  | OR | 95% LB | 95% UB | OR | 95% LB | 95% UB | OR | 95% LB | 95% UB | OR | 95% LB | 95% UB |
|  |  |  |  |  |  |  |  |  |  |  |  |  |  |
| age |  | **1.234** | **1.232** | **1.237** | 1.277 | 1.274 | 1.279 | 1.282 | 1.280 | 1.285 | **1.330** | **1.327** | **1.333** |
| Sex | Boys (ref) |  |  |  |  |  |  |  |  |  |  |  |  |
|  | Girls | 1.097 | 1.090 | 1.104 | 1.113 | 1.107 | 1.120 | 1.081 | 1.074 | 1.088 | 1.089 | 1.082 | 1.097 |
| School Type | Primary School (ref) |  |  |  |  |  |  |  |  |  |  |  |  |
|  | Secondary School | **1.106** | **1.094** | **1.119** | 0.864 | 0.855 | 0.873 | 0.781 | 0.772 | 0.790 | **0.707** | **0.699** | **0.716** |
|  |  |  |  |  |  |  |  |  |  |  |  |  |  |
| age |  | 1.250 | 1.248 | 1.252 | 1.253 | 1.251 | 1.255 | 1.240 | 1.239 | 1.242 | 1.267 | 1.265 | 1.268 |
| Sex | Boys (ref) |  |  |  |  |  |  |  |  |  |  |  |  |
|  | Girls | 1.102 | 1.095 | 1.110 | 1.115 | 1.109 | 1.122 | 1.080 | 1.073 | 1.087 | 1.088 | 1.081 | 1.096 |
| School District | Central and Western (ref) |  |  |  |  |  |  |  |  |  |  |  |  |
|  | Wan Chai | 0.912 | 0.891 | 0.933 | 0.922 | 0.901 | 0.944 | 0.957 | 0.934 | 0.981 | 0.950 | 0.925 | 0.974 |
|  | Eastern | 0.839 | 0.823 | 0.856 | 0.920 | 0.902 | 0.939 | 1.079 | 1.055 | 1.103 | 1.059 | 1.035 | 1.084 |
|  | Southern | 0.802 | 0.781 | 0.823 | 0.788 | 0.768 | 0.807 | 0.837 | 0.816 | 0.859 | 0.846 | 0.824 | 0.870 |
|  | Yau Tsim Mong | 0.826 | 0.808 | 0.844 | 0.842 | 0.824 | 0.861 | 0.912 | 0.890 | 0.934 | 0.969 | 0.945 | 0.994 |
|  | Sham Shui Po | 0.705 | 0.690 | 0.721 | 0.735 | 0.719 | 0.750 | 0.848 | 0.829 | 0.867 | 0.919 | 0.898 | 0.941 |
|  | Kowloon City | 0.923 | 0.906 | 0.942 | 0.900 | 0.883 | 0.917 | 0.910 | 0.891 | 0.929 | 0.960 | 0.939 | 0.982 |
|  | Wong Tai Sin | 0.681 | 0.668 | 0.695 | 0.688 | 0.674 | 0.702 | 0.861 | 0.842 | 0.881 | 0.993 | 0.970 | 1.017 |
|  | Kwun Tong | 0.643 | 0.630 | 0.656 | 0.698 | 0.684 | 0.712 | 0.840 | 0.822 | 0.859 | 0.918 | 0.898 | 0.939 |
|  | Tsuen Wan | 0.724 | 0.707 | 0.741 | 0.742 | 0.725 | 0.759 | 0.925 | 0.902 | 0.948 | 1.033 | 1.006 | 1.060 |
|  | Tuen Mun | 0.617 | 0.605 | 0.630 | 0.626 | 0.614 | 0.639 | 0.723 | 0.707 | 0.739 | 0.768 | 0.751 | 0.786 |
|  | Yuen Long | 0.580 | 0.568 | 0.591 | 0.685 | 0.672 | 0.698 | 0.857 | 0.840 | 0.876 | 0.799 | 0.782 | 0.817 |
|  | North | 0.622 | 0.609 | 0.635 | 0.623 | 0.610 | 0.636 | 0.725 | 0.708 | 0.741 | 0.757 | 0.739 | 0.775 |
|  | Tai Po | 0.594 | 0.581 | 0.608 | 0.625 | 0.611 | 0.639 | 0.718 | 0.701 | 0.736 | 0.735 | 0.717 | 0.754 |
|  | Sai Kung | 0.724 | 0.707 | 0.741 | 0.747 | 0.732 | 0.764 | 0.956 | 0.934 | 0.978 | 1.017 | 0.993 | 1.042 |
|  | Sha Tin | 0.737 | 0.723 | 0.752 | 0.746 | 0.731 | 0.760 | 0.821 | 0.804 | 0.839 | 0.857 | 0.839 | 0.877 |
|  | Kwai Tsing | 0.711 | 0.696 | 0.726 | 0.716 | 0.701 | 0.731 | 0.871 | 0.852 | 0.891 | 0.946 | 0.924 | 0.968 |
|  | Islands | 0.614 | 0.591 | 0.638 | 0.678 | 0.658 | 0.698 | 0.766 | 0.742 | 0.791 | 0.728 | 0.703 | 0.753 |
|  |  |  |  |  |  |  |  |  |  |  |  |  |  |
| age |  | **0.954** | **0.950** | **0.958** | 1.018 | 1.013 | 1.023 | 1.054 | 1.048 | 1.060 | **1.095** | **1.088** | **1.102** |
| Sex | Boys (ref) |  |  |  |  |  |  |  |  |  |  |  |  |
|  | Girls | 1.093 | 1.086 | 1.100 | 1.108 | 1.102 | 1.115 | 1.077 | 1.070 | 1.084 | 1.086 | 1.079 | 1.094 |
| Grade of Student | P1 (ref) |  |  |  |  |  |  |  |  |  |  |  |  |
|  | P2 | 1.475 | 1.455 | 1.496 | 1.501 | 1.479 | 1.523 | 1.492 | 1.468 | 1.516 | 1.528 | 1.504 | 1.553 |
|  | P3 | 2.151 | 2.117 | 2.185 | 2.127 | 2.091 | 2.163 | 2.046 | 2.008 | 2.084 | 2.106 | 2.066 | 2.147 |
|  | P4 | 3.088 | 3.029 | 3.148 | 2.861 | 2.803 | 2.919 | 2.638 | 2.580 | 2.698 | 2.770 | 2.706 | 2.837 |
|  | P5 | 4.292 | 4.194 | 4.393 | 3.594 | 3.509 | 3.683 | 3.079 | 2.997 | 3.162 | 3.243 | 3.151 | 3.338 |
|  | P6 | 5.768 | 5.613 | 5.927 | 4.409 | 4.285 | 4.536 | 3.660 | 3.545 | 3.778 | 3.761 | 3.634 | 3.893 |
|  | S1 | 7.590 | 7.352 | 7.836 | 5.109 | 4.942 | 5.281 | 3.880 | 3.739 | 4.026 | 3.812 | 3.661 | 3.968 |
|  | S2 | 9.136 | 8.809 | 9.475 | 5.667 | 5.456 | 5.886 | 4.156 | 3.984 | 4.336 | 3.894 | 3.718 | 4.077 |
|  | S3 | 10.671 | 10.235 | 11.126 | 6.232 | 5.970 | 6.505 | 4.272 | 4.074 | 4.479 | 3.782 | 3.591 | 3.984 |
|  | S4 | **11.923** | **11.370** | **12.504** | 6.699 | 6.387 | 7.026 | 4.650 | 4.415 | 4.897 | **4.025** | **3.802** | **4.262** |
|  |  |  |  |  |  |  |  |  |  |  |  |  |  |
| age |  | 1.251 | 1.249 | 1.252 | 1.253 | 1.252 | 1.255 | 1.240 | 1.238 | 1.242 | 1.266 | 1.265 | 1.268 |
| Sex | Boys (ref) |  |  |  |  |  |  |  |  |  |  |  |  |
|  | Girls | 1.102 | 1.095 | 1.109 | 1.116 | 1.109 | 1.123 | 1.080 | 1.073 | 1.087 | 1.088 | 1.080 | 1.095 |
| Student Health Service Centre | Chai Wan (ref) |  |  |  |  |  |  |  |  |  |  |  |  |
|  | Kowloon Bay | 0.875 | 0.861 | 0.889 | 0.843 | 0.830 | 0.856 | 0.801 | 0.788 | 0.814 | 0.869 | 0.854 | 0.884 |
|  | Kowloon City LC | 1.053 | 1.037 | 1.068 | 0.965 | 0.951 | 0.979 | 0.866 | 0.853 | 0.880 | 0.938 | 0.922 | 0.953 |
|  | Lam Tin | 0.835 | 0.822 | 0.847 | 0.820 | 0.808 | 0.832 | 0.891 | 0.877 | 0.904 | 0.971 | 0.956 | 0.987 |
|  | South Kwai Chung | 0.854 | 0.841 | 0.867 | 0.801 | 0.790 | 0.813 | 0.843 | 0.830 | 0.856 | 0.929 | 0.914 | 0.944 |
|  | Sha Tin | 0.883 | 0.870 | 0.897 | 0.818 | 0.805 | 0.830 | 0.780 | 0.768 | 0.793 | 0.834 | 0.820 | 0.849 |
|  | Tai Po | 0.708 | 0.695 | 0.721 | 0.700 | 0.688 | 0.713 | 0.682 | 0.668 | 0.696 | 0.711 | 0.696 | 0.726 |
|  | Shek Wu Hui | 0.750 | 0.737 | 0.763 | 0.685 | 0.674 | 0.697 | 0.691 | 0.679 | 0.704 | 0.739 | 0.725 | 0.753 |
|  | Tuen Mun | 0.736 | 0.724 | 0.747 | 0.690 | 0.679 | 0.700 | 0.689 | 0.677 | 0.701 | 0.748 | 0.734 | 0.762 |
|  | Western | 1.088 | 1.072 | 1.105 | 0.987 | 0.973 | 1.002 | 0.872 | 0.858 | 0.886 | 0.900 | 0.884 | 0.915 |
|  | TWS Wu York Yu | 0.811 | 0.798 | 0.824 | 0.761 | 0.750 | 0.772 | 0.809 | 0.796 | 0.822 | 0.961 | 0.944 | 0.978 |
|  | Yuen Long | 0.691 | 0.680 | 0.702 | 0.754 | 0.743 | 0.765 | 0.815 | 0.802 | 0.828 | 0.775 | 0.761 | 0.788 |
|  |  |  |  |  |  |  |  |  |  |  |  |  |  |
| age |  | 1.232 | 1.230 | 1.234 | 1.224 | 1.222 | 1.225 | 1.208 | 1.207 | 1.210 | 1.233 | 1.231 | 1.235 |
| Sex | Boys (ref) |  |  |  |  |  |  |  |  |  |  |  |  |
|  | Girls | 1.103 | 1.096 | 1.110 | 1.116 | 1.109 | 1.123 | 1.080 | 1.073 | 1.087 | 1.088 | 1.080 | 1.095 |
| Type of Housing | Public rental housing (ref) |  |  |  |  |  |  |  |  |  |  |  |  |
|  | Subsidized home ownership flats | **1.217** | **1.204** | **1.230** | 1.202 | 1.190 | 1.215 | 1.115 | 1.102 | 1.128 | **1.078** | **1.064** | **1.092** |
|  | Private Housing | **1.341** | **1.330** | **1.352** | 1.288 | 1.278 | 1.297 | 1.080 | 1.071 | 1.089 | **0.990** | **0.981** | **0.998** |
|  | Villas/Bungalows/Modern Village Houses | **0.767** | **0.751** | **0.782** | 0.762 | 0.748 | 0.776 | 0.743 | 0.729 | 0.756 | **0.720** | **0.707** | **0.734** |
|  | Squatter/Temp. Housing Area/Stone Hut | **0.561** | **0.545** | **0.578** | 0.651 | 0.628 | 0.674 | 0.685 | 0.654 | 0.717 | **0.713** | **0.672** | **0.757** |
|  |  |  |  |  |  |  |  |  |  |  |  |  |  |
| age |  | 1.250 | 1.249 | 1.252 | 1.253 | 1.252 | 1.255 | 1.240 | 1.238 | 1.241 | 1.263 | 1.261 | 1.265 |
| Sex | Boys (ref) |  |  |  |  |  |  |  |  |  |  |  |  |
|  | Girls | 1.101 | 1.094 | 1.108 | 1.115 | 1.108 | 1.122 | 1.081 | 1.073 | 1.088 | 1.088 | 1.081 | 1.096 |
| Home District | Central & Western (ref) |  |  |  |  |  |  |  |  |  |  |  |  |
|  | Wan Chai | 0.957 | 0.920 | 0.995 | 0.931 | 0.902 | 0.962 | 0.951 | 0.919 | 0.985 | 0.960 | 0.925 | 0.996 |
|  | Eastern | 0.926 | 0.911 | 0.943 | 0.992 | 0.973 | 1.012 | 1.099 | 1.075 | 1.123 | 1.058 | 1.032 | 1.084 |
|  | Southern | 0.898 | 0.875 | 0.921 | 0.877 | 0.857 | 0.899 | 0.922 | 0.898 | 0.946 | 0.938 | 0.911 | 0.965 |
|  | Yau Tsim Mong | 0.885 | 0.865 | 0.904 | 0.885 | 0.864 | 0.906 | 0.900 | 0.877 | 0.923 | 0.935 | 0.909 | 0.961 |
|  | Sham Shui Po | 0.781 | 0.765 | 0.797 | 0.782 | 0.765 | 0.799 | 0.909 | 0.888 | 0.931 | 0.941 | 0.917 | 0.966 |
|  | Kowloon City | 0.964 | 0.945 | 0.983 | 0.970 | 0.950 | 0.991 | 0.979 | 0.956 | 1.002 | 1.019 | 0.993 | 1.045 |
|  | Wong Tai Sin | 0.757 | 0.743 | 0.771 | 0.750 | 0.735 | 0.765 | 0.895 | 0.874 | 0.916 | 1.011 | 0.986 | 1.038 |
|  | Kwun Tong | 0.746 | 0.734 | 0.759 | 0.771 | 0.756 | 0.786 | 0.894 | 0.874 | 0.913 | 0.933 | 0.911 | 0.955 |
|  | Tsuen Wan | 0.809 | 0.793 | 0.826 | 0.814 | 0.796 | 0.833 | 0.966 | 0.941 | 0.991 | 1.009 | 0.982 | 1.038 |
|  | Tuen Mun | 0.667 | 0.656 | 0.679 | 0.667 | 0.654 | 0.681 | 0.757 | 0.740 | 0.775 | 0.804 | 0.783 | 0.824 |
|  | Yuen Long | 0.627 | 0.617 | 0.638 | 0.714 | 0.701 | 0.728 | 0.867 | 0.849 | 0.886 | 0.818 | 0.799 | 0.838 |
|  | North | 0.679 | 0.666 | 0.692 | 0.663 | 0.649 | 0.677 | 0.748 | 0.731 | 0.766 | 0.829 | 0.807 | 0.851 |
|  | Tai Po | 0.653 | 0.640 | 0.666 | 0.676 | 0.661 | 0.691 | 0.757 | 0.738 | 0.776 | 0.761 | 0.740 | 0.783 |
|  | Sai Kung | 0.787 | 0.772 | 0.803 | 0.811 | 0.795 | 0.828 | 0.965 | 0.943 | 0.987 | 1.001 | 0.976 | 1.026 |
|  | Sha Tin | 0.815 | 0.801 | 0.829 | 0.803 | 0.788 | 0.819 | 0.860 | 0.842 | 0.879 | 0.894 | 0.872 | 0.915 |
|  | Kwai Tsing | 0.773 | 0.758 | 0.787 | 0.757 | 0.742 | 0.772 | 0.870 | 0.851 | 0.890 | 0.932 | 0.909 | 0.955 |
|  | Islands | 0.707 | 0.680 | 0.734 | 0.721 | 0.701 | 0.741 | 0.773 | 0.749 | 0.796 | 0.734 | 0.710 | 0.758 |
|  |  |  |  |  |  |  |  |  |  |  |  |  |  |
| LB, lower bound; UB, upper bound; SHS, Student Health Service | | | | | | | | | | | | | |
| School year 2009/10 was excluded because SHS limited the annual appointments in that year for taking part in the Human Swine Influenza Vaccination Programme | | | | | | | | | | | | | |

**Supplementary Table 7.** Odds ratios for reduced visual acuity on the schoolchildren living in China relative to Central & Western District in Hong Kong from 2013/14 to 2016/17.

|  |  | Univariate logistic analysis | | |  | Univariate logistic analysis | | |  |  |  |  |
| --- | --- | --- | --- | --- | --- | --- | --- | --- | --- | --- | --- | --- |
| School |  | before adjusting for age and sex | | |  | after adjusting for age and sex | | |  | Multivariate logistic analysis | | |
| Year |  | Estimate | 95% LB | 95% UB |  | Estimate | 95% LB | 95% UB |  | Estimate | 95% LB | 95% UB |
|  |  |  |  |  |  |  |  |  |  |  |  |  |
| 2013/14 |  | 0.278 | 0.259 | 0.298 |  | 0.439 | 0.408 | 0.471 |  | 0.499 | 0.447 | 0.557 |
| 2014/15 |  | 0.290 | 0.272 | 0.309 |  | 0.457 | 0.428 | 0.488 |  | 0.484 | 0.437 | 0.537 |
| 2015/16 |  | 0.321 | 0.302 | 0.341 |  | 0.492 | 0.463 | 0.523 |  | 0.497 | 0.450 | 0.549 |
| 2016/17 |  | 0.342 | 0.323 | 0.362 |  | 0.507 | 0.478 | 0.538 |  | 0.477 | 0.433 | 0.525 |
|  |  |  |  |  |  |  |  |  |  |  |  |  |
| LB, lower bound; UB, upper bound | | | | | | | | | | | | |

**Supplementary Table 8.** The proportion of live births whose parents are not Hong Kong Permanent Residents to the total number of live births from 2001 to 2017.

| **Reference period** | **Total number of live births** | **Live births whose parents are not Hong Kong Permanent Residents** | **Proportion (%)** |
| --- | --- | --- | --- |
| 2001 | 48219 | 620 | 1.29 |
| 2002 | 48209 | 1250 | 2.6 |
| 2003 | 46965 | 2070 | 4.41 |
| 2004 | 49796 | 4102 | 8.24 |
| 2005 | 57098 | 9273 | 16.25 |
| 2006 | 65626 | 16044 | 24.45 |
| 2007 | 70875 | 18816 | 26.55 |
| 2008 | 78822 | 25269 | 32.06 |
| 2009 | 82095 | 29766 | 36.26 |
| 2010 | 88584 | 32653 | 36.87 |
| 2011 | 95451 | 35736 | 37.44 |
| 2012 | 91558 | 26715 | 29.18 |
| 2013 | 57084 | 790 | 1.39 |
| 2014 | 62305 | 823 | 1.33 |
| 2015 | 59878 | 775 | 1.3 |
| 2016 | 60856 | 606 | 1 |
| 2017 | 56548 | 502 | 0.89 |
| Source: Fertility Trend in Hong Kong, 1981 to 2017, Hong Kong Monthly Digest of Statistics December 2018, Demographic Statistics Section, Census and Statistics Department, HKSAR Government (https://www.statistics.gov.hk/pub/B71812FA2018XXXXB0100.pdf) | | | |
